# The role of catastrophizing and basic psychological needs satisfaction on health-related quality of life and pain in patients with lumbar disc herniation

**DOI:** 10.3389/fpsyg.2023.1147254

**Published:** 2023-06-22

**Authors:** Daniela Ionescu, Claudia Iuliana Iacob, Felix Mircea Brehar, Eugen Avram

**Affiliations:** ^1^Department of Sociology, National School of Political and Administrative Studies, Bucharest, Romania; ^2^Laboratory of Health Psychology and Clinical Neuropsychology, Department of Applied Psychology and Psychotherapy, Faculty of Psychology and Educational Sciences, University of Bucharest, Bucharest, Romania; ^3^Department of Neurosurgery, Carol Davila University of Medicine and Pharmacy, Bucharest, Romania

**Keywords:** lumbar disc herniation, pain, quality of life, pain catastrophizing, basic psychological needs satisfaction

## Abstract

**Introduction:**

Lumbar disc herniation (LDH) is one of the most common conditions associated with functional disability, affecting patients’ quality of life (QOL). Disability can be affected by cognitive factors, such as pain catastrophizing. Similarly, unfulfilled basic psychological needs (i.e., autonomy, competence, relatedness) are associated with biases in pain perception and QOL. Using the fear-avoidance model and the self-determination theory, this study investigates: (1) the separate contribution of pain-related variables and basic psychological needs satisfaction in predicting QOL in patients proposed for LDH surgery; (2) pre- and post-surgical differences in pain catastrophizing and basic psychological needs satisfaction.

**Methods:**

First, we used hierarchical regression on 193 patients (M_age_ = 46.10, SD_age_ = 11.40) to identify predictors of QOL. Second, we performed paired *t*-tests on 55 patients to investigate pre- and post-surgical differences in pain catastrophizing and basic psychological needs satisfaction.

**Results:**

Hierarchical regression showed that the model predicts 27% of the variance in QOL; medium pain level, age, pain catastrophizing, and basic psychological needs satisfaction were significant predictors. Also, pain catastrophizing significantly decreased after surgery [t (54) = 6.07, *p* < 0.001, Cohen’s *d* = 0.81], but basic psychological needs satisfaction did not modify significantly.

**Discussion:**

This research confirms the importance of pain perception and pain catastrophizing for LDH patients’ QOL and broadens the applicability of the self-determination theory for spinal patients.

## 1. Introduction

A herniated disc affects around 5–20 individuals per 1,000 each year, and it is most prevalent in persons in their third to fifth decades of life, with a male to female ratio of 2:1 (Fjeld et al., 2019). The most widespread causes for spine surgery in adults are lumbar disc herniations (Schroeder et al., 2016), mainly due to pain and functional disability, both pre- and post-surgical (Hannibal and Bishop, 2014). Pain related to lumbar disc herniation (LDH) can be located in different areas, depending on the placement of the herniated disc. Commonly, patients report sharp, piercing, or radiating leg pain from the damage to the sciatic nerve, low back, buttocks, or foot pain. Additionally, neurological symptoms appear (e.g., numbness, weakness in limbs; Dydyk et al., 2020).

All these manifestations lead to functional disability or impairments in accomplishing basic activities of daily living (ADL; e.g., walking, running, bending, lifting) and instrumental activities of daily living (IADL; e.g., shopping, housekeeping, driving the car; Liu et al., 2020). Furthermore, LDH is associated with poor psychological health and lower quality of life (Falla et al., 2016; Inoue et al., 2017; Arendt-Nielsen et al., 2018; Strøm et al., 2018). In addition, hospitalization costs increase, which makes LDH management a problem of social and economic importance (Angst et al., 2017; Robinson et al., 2017; Hingert et al., 2019). As such, patients need to address the LDH to have better quality of life (QOL), and healthcare research needs to find the most effective ways to manage this condition.

Quality of life is influenced by a series of individual factors, such as depression, anxiety, pain catastrophizing, low mental well-being, dysfunctional coping strategies like rumination (Pierik et al., 2016; Pinto et al., 2018), subjective perception of pain (Shipton, 2011), attitudes toward pain, and personality (Wade and Price, 2000; Costa et al., 2012; da Silva et al., 2017). Such phenomenon are relevant for a patient’s recovery and occur in nearly one third of LDH patients referred for surgery (Archer et al., 2011; Lerman et al., 2015; Dorow et al., 2016, 2017; Chapman and Vierck, 2017; Kim et al., 2018; McNeil et al., 2018; Strøm et al., 2018). Pain and psychological risk factors potentiate each other and affect the patient’s functionality, QOL, and mental well-being both pre-surgery and post-surgery (Anderson et al., 2015; Falla et al., 2016; Arendt-Nielsen et al., 2018; Miller et al., 2018).

Current and past pain intensity are significant predictors of recovery from an acute pain episode after the surgical intervention for LDH (Heymans et al., 2010; Williams et al., 2014). Studies also reported that post-surgical persistent pain was predicted by psychological variables and not by immediate post-surgical acute pain (Horn-Hofmann et al., 2018). The importance of pain catastrophizing, anxiety, depression (Miedema et al., 2016; Alodaibi et al., 2018), and general emotional status in post-surgical pain, functional disability, and patient recovery is well-documented (Campbell et al., 2013; Brox, 2014; Ramírez-Maestre et al., 2014; Rebbeck et al., 2015; Falla et al., 2016; Miller et al., 2018; Pinto et al., 2018). For example, a meta-analysis showed that pain catastrophizing, depression, anxiety, and negative emotions were predictors of acute post-surgical pain, with pain catastrophizing being the strongest predictor (Sobol-Kwapinska et al., 2016). Building upon these results, the current investigation will replicate them by examining the relationship between pain severity, catastrophizing and quality of life in a sample of Romanian patients.

Researchers have used different models to identify risk factors for low QOL and pain in patients with LDH. For example, the Fear Avoidance Model (Vlaeyen and Linton, 2000) explains the transition from acute to chronic pain, where anxiety and pain catastrophizing are important risk factors (Keefe et al., 2004; Campbell et al., 2013; Zale and Ditre, 2015; Hallegraeff et al., 2020). According to this model, fear of pain and pain catastrophizing activate coping mechanisms focused on avoiding potentially painful experiences. In the long-term, this behavior could lead to motor dysfunction, anxiety, and depression, thus amplifying pain, disability (Ramírez-Maestre et al., 2014; Bunzli et al., 2017; Alodaibi et al., 2018) and lowering QOL. In recent years, Riggenbach et al. (2019) explained chronic pain in youth using the Self-Determination Theory (SDT; Deci and Ryan, 2000). SDT claims that three basic psychological needs (BPN) are the foundations of human well-being: autonomy (personal independence, choice, freedom), competence (the feeling that one is able to do what one desires), and relatedness (connection with others). The satisfaction of these BPN was connected to a lower risk for psychopathology, whereas the frustration of these BPN was associated with maladaptive behaviors, such as avoiding pain and functional disability (Riggenbach et al., 2021). However, this model has only been used on young patients with chronic pain of different origins. Given that LDH mainly affects adults and the elderly, we want to see the contribution of BPN in predicting quality of life, in addition to pain.

Regarding spinal patients, the Fear Avoidance Model and the SDT become interconnected and complement each other in several ways. First of all, the SDT promotes autonomy and activity engagement in pain management as ways to diminish fear-related behaviors. Second, the sense of competence enhances patients’ motivation to engage in optimal pain management behavior (Sheldon and Filak, 2008) instead of avoidance. Both frameworks recognize the significance of social support and connection. The Fear Avoidance Model suggests that individuals’ pain-related beliefs can lead to social isolation from family and friends (Sawchuk and Mayer, 2008), while Self-Determination Theory emphasizes the need for relatedness to foster motivation and well-being in life (Deci and Ryan, 2000).

As such, following the Fear Avoidance Model and the SDT, this study has two aims: (1) to examine the contribution of pain-related variables and BPN satisfaction in predicting QOL in adult patients proposed for LDH surgery; (2) to investigate the pre-surgical and post-surgical differences in pain catastrophizing and BPN satisfaction in patients undergoing LDH surgery. We expect patients to have lower levels of pain catastrophizing after surgery, in line with previous studies. Also, exploratory, we anticipate that BPN satisfaction will significantly increase after surgery when patients have good prospects of resuming their daily activities shortly.

## 2. Methods

### 2.1. Participants and design

This study was conducted on a sample of patients with intense back pain. The sample consisted of 193 adult patients with LDH, hospitalized, and proposed for surgical intervention at the “Bagdasar-Arseni” Emergency Hospital in Bucharest, Romania, before the COVID-19 pandemic. Using a cross-sectional design, the participants were assessed before surgery and had a mean age of 46.10, with SD_age_ = 11.40. The gender distribution was relatively balanced, with 102 females (53%) and 85 males (44%). Six participants did not disclose their gender. Most participants were employed. A subsample of 55 patients was also assessed after surgery with pain and BPN satisfaction measures. They had similar gender characteristics as the main sample: 28 females (51%) and 27 males (49.%), with M_age_ = 51.29 (SD_age_ = 1.33). We excluded patients who met one or more of the following criteria: (1) had major surgical interventions in the past, (2) had a terminal illness, (3) had other chronic pain conditions, (3) had a psychiatric diagnosis, (4) had a disability certificate.

### 2.2. Procedure

After hospital admission, the patients with intense back pain were asked to participate in this study. Each patient eligible for inclusion was informed about the study’s aims and gave informed consent for participation. The study followed the principles of the Declaration of Helsinki. All the participants presented pain related to their undergoing clinical condition; therefore, they were fully assisted by a researcher specialized in clinical psychology as they completed the measures. A subsample of 55 patients also completed pain catastrophizing and BPN satisfaction measures after LHD surgery. The process can be visualized in the Supplementary Figure S1 included in the Supplementary material file. The participation was voluntary, no incentives were provided, and the patients could withdraw at any moment without consequences.

### 2.3. Measures

#### 2.3.1. Quality of life

Quality of life perception was assessed with EQ-5D-5 L (EuroQol Group, 2019), which is designed as a general measure for health-related quality of life. The scale has five dimensions, but we used only the three dimensions that assess functional disability in the following areas: mobility, self-care, daily life activities. We chose these dimensions as they are the most relevant when it comes to functional impairment in patients with intense lower back pain (Kose and Hatipoglu, 2012). The items were ranked from 1 (*I have no problems…*) to 5 (*I am unable to…*) in each dimension. For further analysis, we used the sum of scores for the three dimensions. Bigger scores indicated lower QOL. The internal consistency for the three subscales of QOL was acceptable (Cronbach’s *α* = 0.76).

#### 2.3.2. Pain

Pain intensity was assessed using a numeric pain rating scale (NRS; Haefeli and Elfering, 2006). We evaluated pain with two items (medium pain intensity and maximum pain intensity experienced within the last week) ranked on a numeric scale from 0 (no pain) to 10 (maximum level of pain ever felt).

#### 2.3.3. Pain catastrophizing

Pain catastrophizing was investigated with The Pain Catastrophizing Scale (PCS; Sullivan et al., 1995) a measure of catastrophizing thinking related to pain. It contains 13 items (they can be divided into the following subscales: magnification, rumination, and helplessness). The items were rated on a 5-point scale according to their frequency (0 = no catastrophizing thoughts about pain, 4 = these thoughts appear all the time). In the analyses, we used the combined sum score of the PCS (with the range between 0 and 52 points). The internal consistency for the general scale was good (Cronbach’s *α* = 0.93).

#### 2.3.4. Basic psychological needs satisfaction

Basic psychological needs satisfaction was assessed using the Basic Psychological Needs Satisfaction Scale (Deci and Ryan, 2000), a 9 item self-report instrument that investigates the perceived satisfaction of the three basic psychological needs: autonomy, competence, relatedness. The answers were rated on a 5-point Likert scale, from 1 (totally disagree) to 5 (totally agree). On this sample, the scale had a good internal consistency (Cronbach’s *α* = 0.83).

### 2.4. Statistical analysis

We analyzed the data using Jamovi software version 1.19. First, means, standard deviations, and normality indicators were reported, along with the Pearson correlations for all the variables in the study. To test the normality of the distribution, we used the skewness and kurtosis indicators, with acceptable values ranging from −1.96 to +1.96 (George and Mallery, 2011). Next, we reported the p levels for each analysis throughout the study, with *p* < 0.05 being considered the minimum acceptable cutoff. We used hierarchical linear regression to investigate the contribution of the chosen predictors for explaining QOL. The main assumptions of hierarchical regression were met. In the first step, we introduced the demographical variables (age and gender) as control variables. In the following steps, we included medium pain level, pain catastrophizing, maximum pain level and BPN satisfaction, in the order of their descending correlation with QOL. The components of BPN satisfaction were not introduced separately in the regression equations due to concerns of multicollinearity. Finally, we reported unstandardized and standardized B coefficients and standard error for B, confidence intervals, and value of ps. For investigating the pre-surgical and post-surgical differences in pain catastrophizing and BPN satisfaction, we used the paired samples t-test and reported the appropriate indicators (value of *p*, mean difference, confidence intervals, and Cohen’s *d* effect size).

## 3. Results

### 3.1. Descriptive statistics and preliminary analyses

Participants with higher medium and maximum pain levels, more intense pain catastrophizing thoughts, and older age reported lower QOL, as expected. There was no significant correlation between BPN satisfaction and pain levels. For details, please consult Table 1, which also displays the main descriptive statistics, along with the skewness and kurtosis normality indicators. The latter had acceptable values, between −1.96 and + 1.96. None of the variables had missing values, except for age, which had 12 missing values.

**Table 1:** Pearson correlations and descriptive statistics.

	1	2	3	4	5	6	7	8	9
*N* = 193 (pre-surgery)									
1	–								
2	0.27***	–							
3	0.40***	0.06	–						
4	0.27***	0.01	0.64***	–					
5	0.37***	0.18*	0.42***	0.37***	–				
6	−0.21**	0.01	−0.10	−0.06	−0.19**	–			
7	−0.17*	0.09	−0.08	−0.09	−0.20**	0.78***	–		
8	−0.15*	−0.04	−0.05	−0.02	−0.13	0.87***	0.54***	–	
9	−0.19**	−0.01	−0.11	−0.05	−0.15*	0.82***	0.45***	0.62***	–
Mean	11.1	46.1	5.95	7.14	23.8	39.3	13.3	13.2	12.8
SD	3.80	11.4	2.40	2.34	12.4	4.47	1.67	1.79	1.90
Skewness	0.38	0.05	−0.32	−1.03	0.11	−0.50	−0.86	−0.75	−0.69
Kurtosis	−0.45	0.02	−0.34	0.96	−0.62	−0.43	0.42	−0.23	0.07
*N* = 55 (post-surgery)									
Mean					21.29	33.44	11.81	10.71	10.91
SD					1.64	1.01	2.64	3.25	2.81
Skewness					0.66	−0.03	−0.78	−0.19	−0.44
Kurtosis					0.12	−1.03	0.18	−0.90	−0.16

1 = quality of life; 2 = age; 3 = medium pain level; 4 = maximum pain level; 5 = pain catastrophizing; 6 = psychological needs satisfaction; 7 = autonomy; 8 = competence; 9 = relatedness. *p < 0.05, **p < 0.01, ***p < 0.001.

In terms of comparisons between patients assessed only pre-surgically (*N* = 138) and the subsample assessed also post-surgically (*N* = 55), we found that the groups were similar in terms of medium [t (107) = 1.24, *p* = 0.19], maximum [t (100) = 0.41, *p* = 0.68] pain levels and catastrophizing [t (99) = 1.30, *p* = 0.19]. At the same time, the following differences were observed: the subsample evaluated also post-surgically was older compared to the former [t (114) = −3.87, *p* < 0.001, Cohen’s *d* = −0.61] and reported lower BPN satisfaction levels [t (67) = 5.69; *p* < 0.001, Cohen’s *d* = 0.99].

### 3.2. Predicting quality of life

For the study’s first aim, a hierarchical linear regression analysis was conducted to evaluate the prediction of quality of life from pain levels, pain catastrophizing, and each BPN component. The predictors were entered in separate steps, in the order of their descending correlations with the criterion. Additionally, the contribution of age and gender was isolated in the first step of the regression. As presented in Table 2, the overall model is significant (*p* < 0.001) and explains 27% of QOL’s variance.

**Table 2:** Hierarchical regression results for quality of life.

	*B*	95% CI for B		SE B	*β*	*R*^2^	Δ*R*^2^	Δ*F*
		LL	UL					
Step 1						0.07***	0.07***	7.53***
Constant	7.02	4.62	9.42	1.21				
Age	0.09	0.04	0.14	0.02	0.27***			
Gender^a^	−0.23	−1.32	0.85	0.55	−0.03			
Step 2						0.22***	0.14***	34.34***
Constant	3.65	1.17	6.13	1.25				
Age	0.08	0.04	0.12	0.02	0.25***			
Gender	−0.09	−1.09	0.91	0.50	−0.01			
Medium pain	0.60	0.40	0.81	0.10	0.38***			
Step 3						0.25***	0.02*	6.41*
Constant	3.30	0.84	5.76	1.24				
Age	0.07	0.03	0.11	0.02	0.22***			
Gender	0.15	−0.85	1.15	0.50	0.02			
Medium pain	0.48	0.25	0.70	0.11	0.30***			
Pain catastrophizing	0.05	0.01	0.10	0.02	0.18*			
Step 4						0.25***	0.00	0.02
Constant	3.21	0.54	5.89	1.35				
Age	0.07	0.03	0.12	0.02	0.22***			
Gender	0.15	−0.85	1.16	0.51	0.02			
Medium pain	0.46	0.19	0.74	0.14	0.30***			
Pain catastrophizing	0.05	0.01	0.10	0.02	0.18*			
Maximum pain	0.02	−0.25	0.30	0.14	0.01			
Step 5						0.27***	0.01*	4.06*
Constant	7.76	2.58	12.94	2.62				
Age	0.07	0.03	0.12	0.02	0.23***			
Gender	0.16	−0.84	1.16	0.50	0.02			
Medium pain	0.46	0.19	0.74	0.13	0.29***			
Pain catastrophizing	0.05	0.004	0.09	0.02	0.16*			
Maximum pain	0.02	−0.25	0.29	0.14	0.01			
BPN satisfaction	−0.11	−0.22	−0.002	0.05	−0.13*			

^a^ 0 = males; 1 = females. *p < 0.05, **p < 0.01, ***p < 0.001.

Step 1 is significant, which means that the control variables significantly contribute to the model [*F* (2, 178) = 7.53, *p* < 0.001, *R*^2^ = 0.07]. In Step 2, we added the medium pain level, which upgraded the model’s prediction power to 22% [Δ*F* (1, 177) = 34.34, *p* < 0.001, Δ*R*^2^ = 0.14]. Medium pain alone explains 14% of QOL’s variance. Pain catastrophizing, the predictor introduced in Step 3, also brings additional explicative value [Δ*F* (1, 176) = 6.41, *p* = 0.01, ΔR^2^ = 0.02]. Maximum pain level included in Step 4 does not bring additional explicative value over the other variables [Δ*F* (1, 175) = 0.02, *p* = 0.87, ΔR^2^ = 0.00]. In contrast, BPN satisfaction inserted in Step 5 brings a small but significant contribution over the other variables [Δ*F* (1, 174) = 4.06, *p* = 0.04, ΔR^2^ = 0.01]. All these results are summarized in Table 2, along with the main predictors for QOL. Additionally, scatterplots for each regression step can be consulted in the Supplementary Figures S2–S6.

As observed in Table 2, the analysis of each predictor within its step shows that medium pain levels, age, pain catastrophizing, and basic psychological needs are significant predictors of QOL. Medium pain level is the strongest predictor (*β* = 0.29, *p* < 0.001 in Step 5). Contrary to expectations, the maximum pain level did not predict QOL (*β* = 0.01, *p* = 0.87 in Step 5) in the current framework. This may be due to the fact that severe low back pain can be episodic (Levi et al., 2018).

### 3.3. Pre-surgical and post-surgical differences in pain catastrophizing and BPN satisfaction

As expected, pain catastrophizing decreased significantly after surgery, with a large effect size [t (54) = 6.07, *p* < 0.001, Cohen’s *d* = 0.81]. BPN satisfaction [t (54) = 1.16, *p* = 0.25] and associated subscales did not change significantly after surgery. This result is probably due to the fact that data were collected shortly after surgery before patients left the hospital and resumed their activities, and BPN satisfaction is a concept that reflects long-term functioning. All the results are detailed in Table 3.

**Table 3:** Pre-surgical and post-surgical differences for LDH patients.

Variables	Mean difference	Std. error mean	95% confidence interval		*t*-value	Cohen’s *d*
			LL	UL		
Pain catastrophizing	8.37	1.38	5.60	11.13	6.07***	0.81
BPN satisfaction	0.58	0.49	−0.41	1.58	1.16	0.15
Autonomy	0.18	1.59	−0.24	0.61	0.87	0.11
Competence	0.19	1.72	−0.26	0.66	0.85	0.11
Relatedness	0.20	2.03	−0.35	0.75	0.73	0.09

*** *p* < 0.001.

## 4. Discussion

This study aimed to investigate: (1) the separate contribution of pain-related variables and BPN satisfaction in predicting QOL of patients with LDH before surgery; (2) the differences in pain catastrophizing and BPN satisfaction before and after LDH surgery. Understanding these interactions can extend the applications of the SDT theory and consolidate the contribution of biopsychosocial factors in LDH patients’ QOL.

The regression analyses showed that medium pain level, pain catastrophizing, BPN satisfaction and age were significant predictors of QOL in the presumed direction. The results reinforce the contribution of the Fear Avoidance Model (Vlaeyen and Linton, 2000) and the SDT (Deci and Ryan, 2000) in explaining QOL in adult patients proposed for LDH surgery. The strongest predictor was the perceived medium pain level, and contrary to expectations, maximum pain level did not predict QOL. This is probably due to the fact that the medium level of pain is associated with a longer pain duration and the maximum level of pain with an outbreak of acute pain, which influences short-term functioning (Levi et al., 2018). Patients report maximum pain as an episode. As such, the pain of medium but subjective intensity is negatively associated with the patient’s daily functional abilities. This urges healthcare researchers to develop more effective treatments addressed to medium, long-term pain levels in spinal patients.

Pain catastrophizing, one of the most prevalent psychological risk factors for pain and disability (Brox, 2014) also predicted QOL, as expected. It increases pain sensitivity via anxiety, sympathetic nervous system predominance (Bandeira et al., 2021) and disruptions in neurophysiological mechanisms of pain inhibition (Toledo et al., 2020). As such, designing pre-operative interventions targeting the catastrophizing thoughts in LDH patients may help them better cope with pain and reduce post-operative recovery time (Burns and Moric, 2011). BPN satisfaction was also a significant predictor of QOL. Even though its contribution to QOL was weaker compared to medium pain, BPN satisfaction matters in understanding the psychological and functional profile of the patient. On the one hand, it provides evidence that this framework is valuable for adult patients, not just for pain struggling youth. Further studies are necessary to establish to what extent rehabilitation programs should include activities designed to improve competence, autonomy, and relatedness, in addition to pain management strategies.

Age predicted QOL and this finding is in line with previous work showing that functional disability levels increase as we grow old due to the natural degenerative processes in the human body and different risk factors (e.g., poverty, overuse, chronic conditions, depression) (Griffith et al., 2010; Saito et al., 2014; Aguiar et al., 2019; Vaish et al., 2020). The degree of wear and tear bring supplementary demand on the human body, becoming harder to perform in daily life and lowering health-related QOL (Gurcay et al., 2010). In addition, different pain levels are associated with lower QOL in older adults, especially severe pain (Makino et al., 2019).

Regarding the second aim of this study, the results showed that patients’ pain catastrophizing scores significantly decreased after surgery, as presumed, but the BPN satisfaction levels did not increase significantly. As stated previously, before their discharge, the patients were still in post-operative recovery and probably could not perceive any differences regarding their autonomy, self-competence, or their social connections with others. The participants’ age could have also impacted this result, since their mean age was 51 years and recovery is usually slower (Mattila et al., 2005). Therefore, future studies interested in the implication of SDT theory for spinal patients should measure BPN satisfaction after patient discharge from the hospital, allowing them to immerse in daily life activities.

As such, the current study assists in this direction by highlighting the contribution of pain-related variables and BPN satisfaction to functional disability aspects of QOL, a major concern for patients with LDH. Nevertheless, it is necessary to discuss the study’s main limitations, especially from a methodological point of view. The cross-sectional design used in the hierarchical regression gives insight on the data at a single point in time, providing no information regarding temporal changes in pain-related variables and QOL. The findings can not be generalized due to the convenience sampling method. This type of sampling method introduces biases in data collection, such as self-selection and sampling bias. However, most findings were in line with existing evidence.

## Data availability statement

The datasets presented in this study can be found in online repositories. The names of the repository/repositories and accession number(s) can be found at: http://doi.org/10.17632/7tgc2bmkw3.1.

## Ethics statement

The studies involving human participants were reviewed and approved by the Ethical Council of Bagdasar-Arseni Emergency Hospital. The patients/participants provided their written informed consent to participate in this study.

## Author contributions

DI and EA contributed to the conception and design of the study. DI, EA, and FB contributed to its implementation. CI performed the statistical analysis. DI and CI wrote the first draft of the manuscript. EA and FB reviewed the manuscript. All authors read and approved the submitted version.

## Conflict of interest

The authors declare that the research was conducted in the absence of any commercial or financial relationships that could be construed as a potential conflict of interest.

## Publisher’s note

All claims expressed in this article are solely those of the authors and do not necessarily represent those of their affiliated organizations, or those of the publisher, the editors and the reviewers. Any product that may be evaluated in this article, or claim that may be made by its manufacturer, is not guaranteed or endorsed by the publisher.
