# Supplementary material for: The role of catastrophizing and basic psychological needs satisfaction on health-related quality of life and pain in patients with lumbar disc herniation
Source: Front Psychol. 2023 Jun 22;14:1147254. doi: 10.3389/fpsyg.2023.1147254 (PMC10323192; doi:10.3389/fpsyg.2023.1147254)
Supplement: Supplementary file 1 [file Data_Sheet_1.PDF]

Supplementary Material

The Role of Catastrophizing and Basic Psychological Needs Satisfaction on Health-related Quality of Life and Pain in Patients with Lumbar Disc Herniation

Daniela Ionescu[1], Claudia Iuliana Iacob[2*], Felix Mircea Brehar[3], Eugen Avram[2]

[1] Department of Sociology, National School of Political and Administrative Studies, Bucharest, Romania

[2]Laboratory of Health Psychology and Clinical Neuropsychology, Department of Applied Psychology and Psychotherapy, Faculty of Psychology and Educational Sciences, University of Bucharest, Romania

[3]Department of Neurosurgery, "Carol Davila" University of Medicine and Pharmacy, Bucharest, Romania

*** Correspondence:** Claudia Iuliana Iacob; e-mail: claudia-iuliana.iacob@fpse.unibuc.ro

1.1 Supplementary Figures

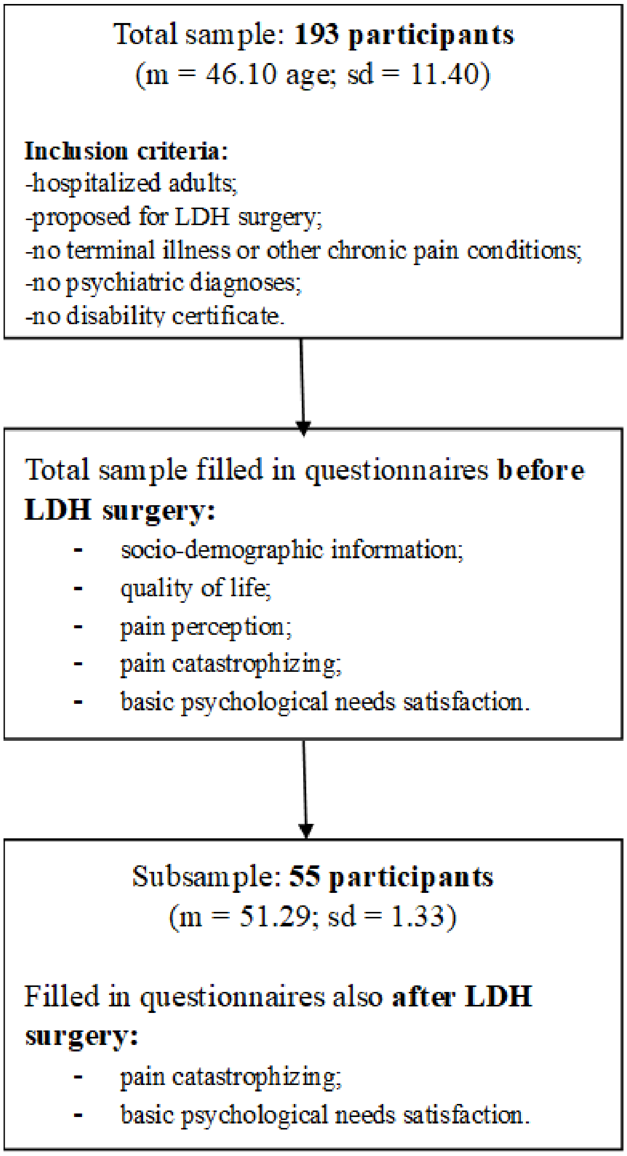

Supplementary Figure 1. Flow chart presenting the study's procedure.

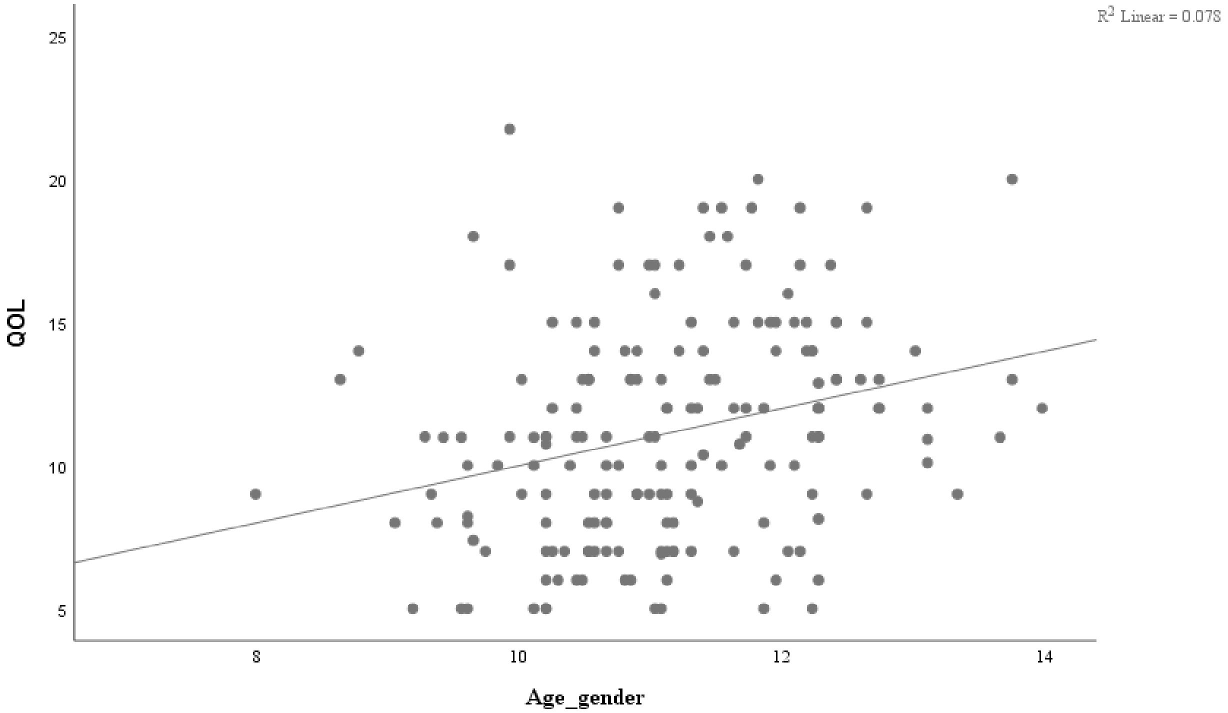

Supplementary Figure 2. Scatterplot depicting the relationship between quality of life (QOL) as criterion and the predictors (age and gender) from Step 1 of the hierarchical regression model.

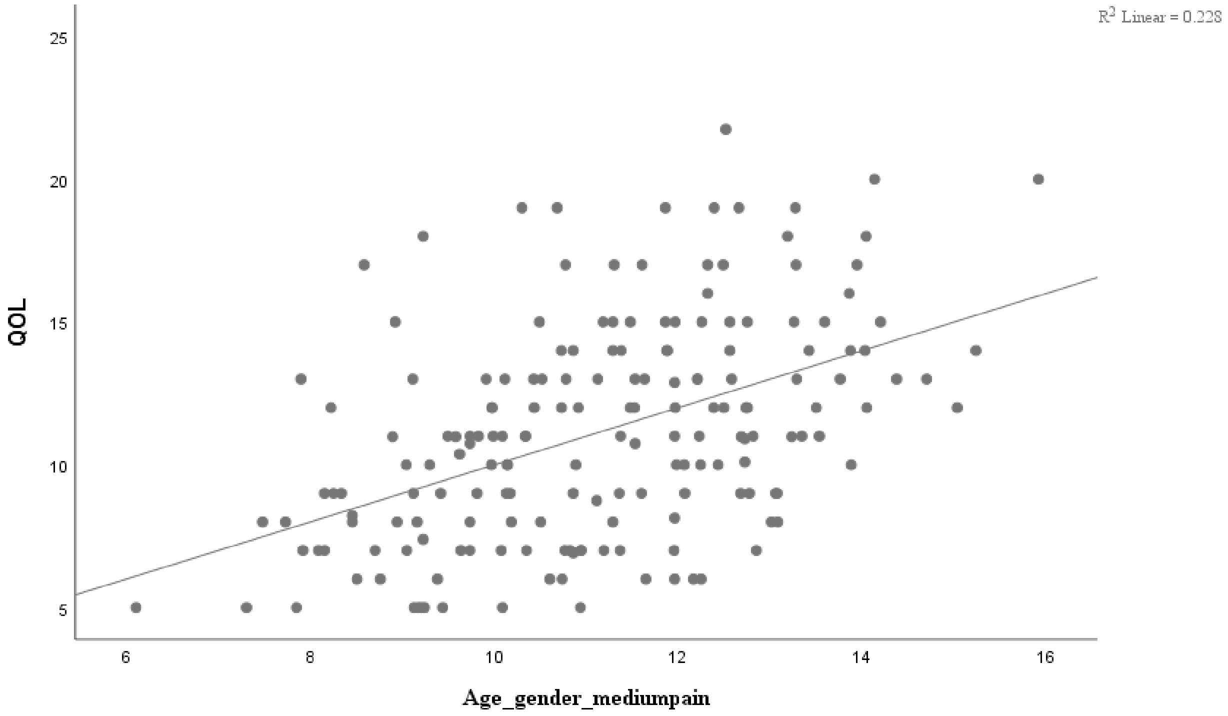

Supplementary Figure 3. Scatterplot depicting the relationship between quality of life (QOL) as criterion and the predictors (age, gender, and medium pain) from Step 2 of the hierarchical regression model.

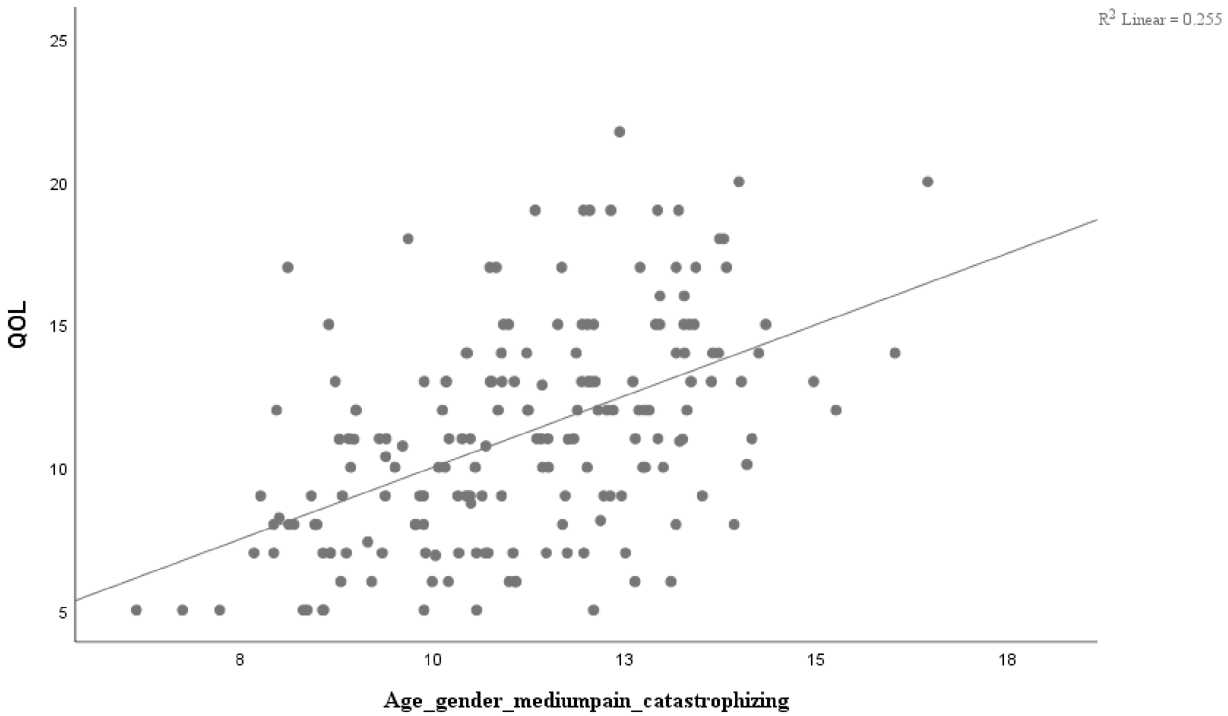

Supplementary Figure 4. Scatterplot depicting the relationship between quality of life (QOL) as criterion and the predictors (age, gender, medium pain and pain catastrophizing) from Step 3 of the hierarchical regression model.

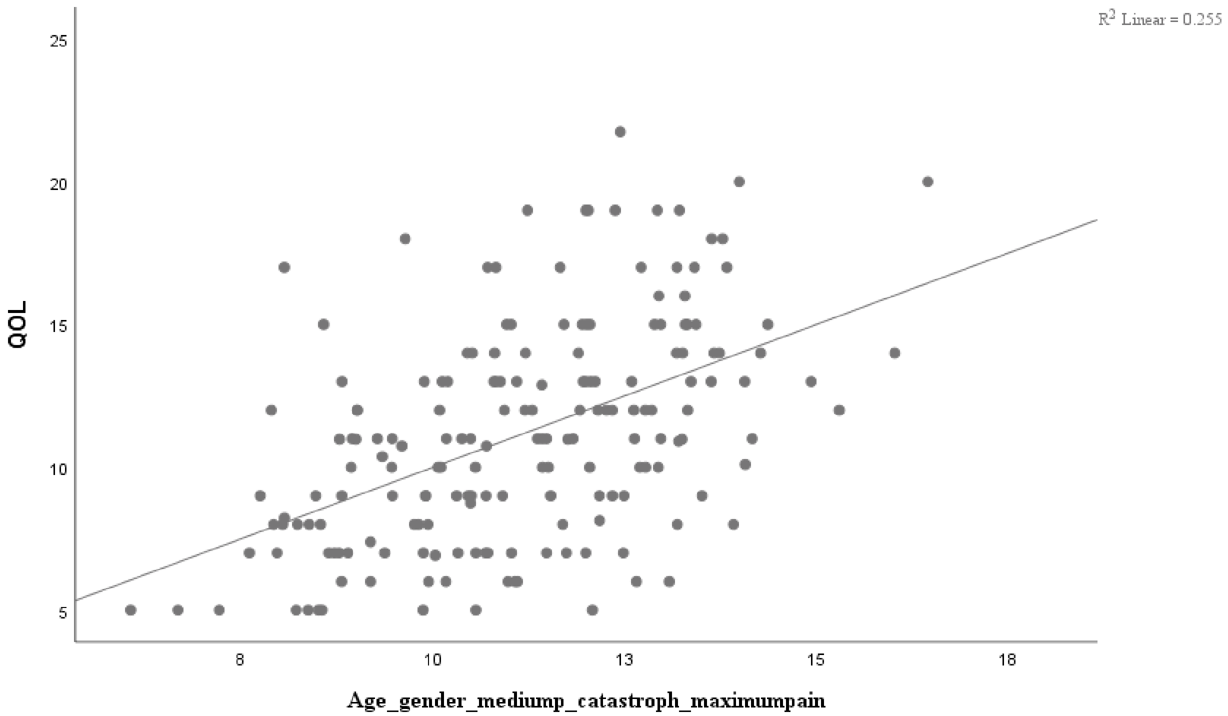

Supplementary Figure 5. Scatterplot depicting the relationship between quality of life (QOL) as criterion and the predictors (age, gender, medium pain, pain catastrophizing and maximum pain) from Step 4 of the hierarchical regression model.

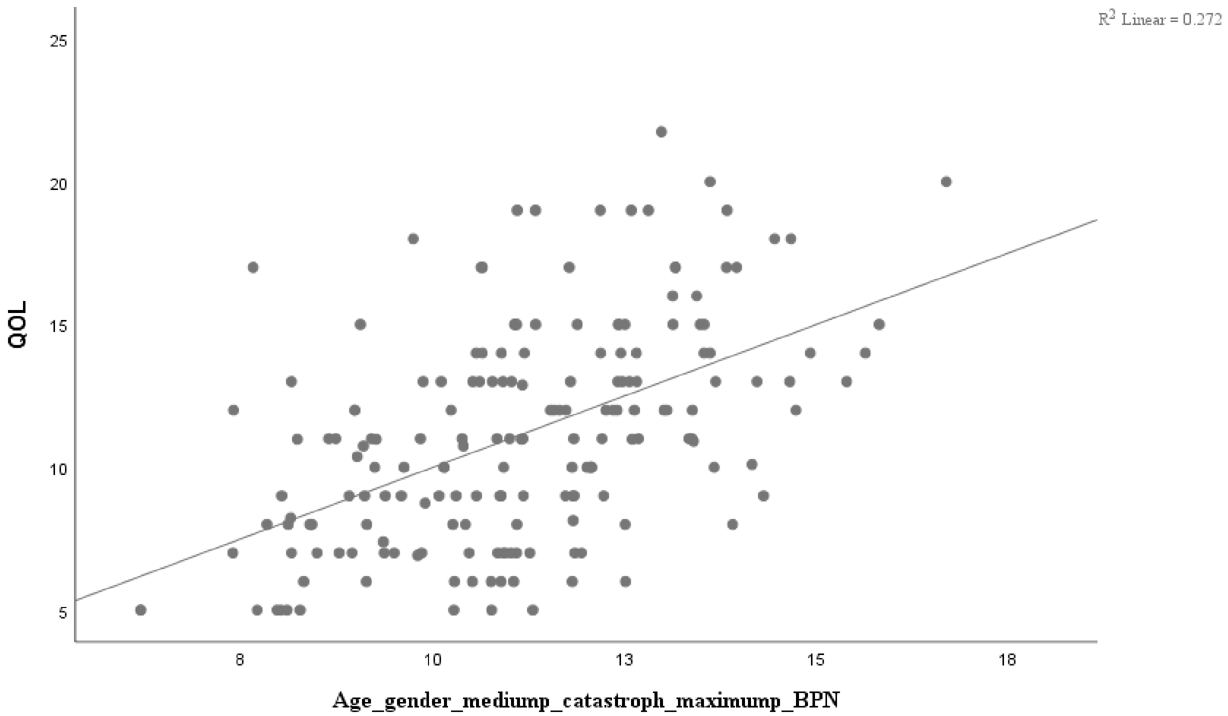

Supplementary Figure 6. Scatterplot depicting the relationship between quality of life (QOL) as criterion and the predictors (age, gender, medium pain, pain catastrophizing, maximum pain and basic psychological needs satisfaction) from Step 5 of the hierarchical regression model.
